# Supplementary material for: Vermont: a multi-perspective visual interactive platform for mutational analysis
Source: BMC Bioinformatics. 2017 Sep 13;18(Suppl 10):403. doi: 10.1186/s12859-017-1789-3 (PMC5606220; doi:10.1186/s12859-017-1789-3)
Supplement: Supplementary file 1 — Supplementary material. Additional details and figures to support on the understanding and usage of VERMONT. http://bioinfo.dcc.ufmg.br/vermont/download/supplementary-material.pdf. (PDF 3070 kb) [file 12859_2017_1789_MOESM1_ESM.pdf]

ADDITIONAL FILE 1

Vermont: a multi-perspective visual interactive platform for mutational analysis

Alexandre V Fassio[1,2*†], Pedro M Martins[1,2†], Samuel da S Guimarães[3], Sócrates S A Junior[3], Vagner S Ribeiro[3], Raquel C de Melo-Minardi[1] and Sabrina de A Silveira[3]

In this document we present additional details and figures about VERMONT platform. The document is organized in sections that are correspondent to those in the main document.

Methods

Input module

Figure 1 shows VERMONT input module.

Topological properties module

Here we comment on some uses of graphs and network measures in a biological context. Bongo[1] uses graphs to represent residue-residue interaction networks and to assign key residues that are important for maintaining such networks. Also, they applied a graph theory concept, vertex cover, which identifies key residues for analyzing structural effects of single point mutations. In [2], complex networks were used to study the role of a residue in local and global structures. High betweenness is expected for key residues that act as a bridge in protein structure, such as those that bring together two different secondary structures. Closeness, in turn, could indicate the functional role of a residue. Also in [2], high closeness values were observed for disease-associated nsSNPs.

In VERMONT, three common complex network centrality measures were computed for each residue. Next, we describe them in detail.

- Degree: the degree of a vertex in a graph is the number of edges connected to it. For an undirected graph of n vertices, the degree k_i of a vertex i can be written in terms of the adjacency matrix as $k_i = \sum_{j=1}^{n} A_{ij}$.
- Betweenness: measures the extent to which a vertex lies on paths between other vertices. Let n_i to be the number of geodesic paths from vertex s to vertex t that pass through vertex i. Let g_{st} to be the total number of geodesic paths from s to t. Then the betweenness centrality of i is $x_i = \sum_{st} \frac{n^i_{st}}{g_{st}}$.
- Closeness: measures the mean distance from a vertex to all other vertices. Let d_i be the length of a geodesic path from i to j, meaning the number of edges along the path. Then the mean geodesic distance from vertex i to vertex j, averaged over all vertices j in the network, is $l_i = \frac{1}{n}\sum_j d_{ij}$. The mean l_i is not a centrality measure since it gives low values for central vertices and high values for less central ones. Therefore, the closeness centrality C_i is the inverse of l_i: $C_i = \frac{1}{l_i}$.

Energy variation prediction

The effects caused by a mutation can be evaluated through the calculation of Gibbs free energy change ($\Delta\Delta G$). Bearing this in mind, we combined energy variation with our visualization modules to potentialize the analysis of specialists. Currently, the prediction of the effect of each mutation is performed with the FoldX tool. To do so, the wild structure was defined as the input, and the FoldX default parameters for pH (7) and temperature (298 K) were used.

Departing from the standard deviation (0.46 kcal/mol) [3], we divided the effects into seven categories in which values range from highly stabilizing ($\Delta\Delta G <$ -1.84 kcal/mol), stabilizing (-1.84 kcal/mol $\leq \Delta\Delta G <$ -0.92 kcal/mol), slightly stabilizing (-0.92 kcal/mol $\leq \Delta\Delta G <$ -0.46 kcal/mol), neutral (-0.46 kcal/mol $< \Delta\Delta G \leq$ 0.46 kcal/mol), slightly destabilizing (+0.46 kcal/mol $< \Delta\Delta G \leq$ +0.92 kcal/mol), destabilizing (+0.92 kcal/mol $< \Delta\Delta G \leq$ +1.84 kcal/mol) and highly destabilizing ($\Delta\Delta G >$ 1.84 kcal/mol).

Results and Discussion

In this section, we use VERMONT to visually analyze 6 disease-associated mutations in tumor suppressor protein, p53, experimentally studied by Fersht and co-workers [4, 5]. We discuss a total of 8 mutations, showed in Table 1, 2 illustrative cases are in the main paper and the other 6 are in the *Additional file 1* due to space limitations. Also, some additional figures for

*Correspondence: alexandrefassio@dcc.ufmg.br
[1] Department of Computer Science, Universidade Federal de Minas Gerais, Belo Horizonte, Brazil
[2] Department of Biochemistry and Immunology, Universidade Federal de Minas Gerais, Belo Horizonte, Brazil
Full list of author information is available at the end of the article
†Equal contributor

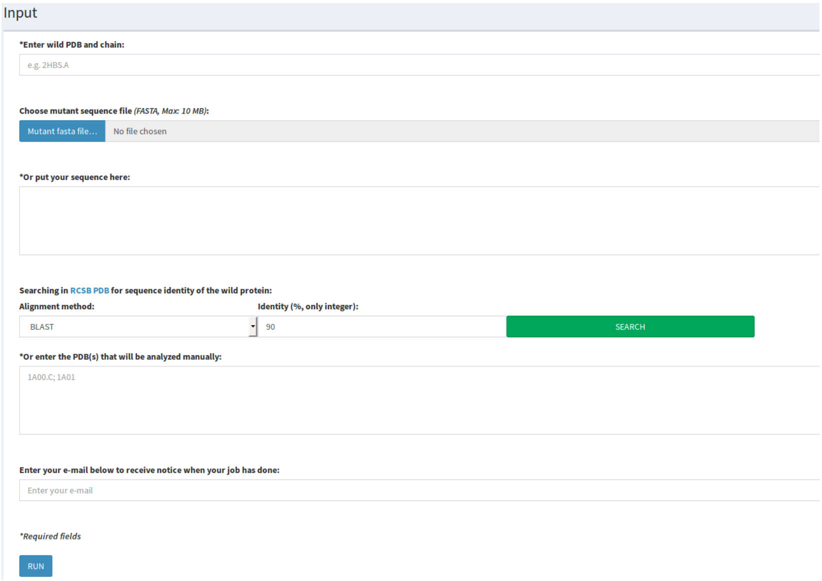

Figure 1 VERMONT *Input* module. It takes as input the PDB id and chain of a wild protein, the FASTA sequence of the respective mutant protein and a set of protein structures similar to the wild protein.

discussions that are in the main paper are showed in this section:

- Figure 2 shows the conservation on alignment position 180 (mutation Arg273His in protein p53) on the *Structure based sequence alignment module.*
- Figure 3 provides the topological properties for mutation Arg273His in protein p53. Alignment position 180, which corresponds to this mutation, is highlighted.
- Figure 4 shows interactions for Arg270 of 3EXL.A, which is in the alignment position 180 (related to Arg273His in protein p53). Interactions are displayed in a 3D molecule viewer and in a 2D graph.
- Figure 5 provides the topological properties for mutation Ile195Thr in protein p53. Alignment position 102, which corresponds to this mutation, is highlighted.

Use case

VERMONT input parameters were (i) PDB id 1TSR.A as wild protein; (ii) the mutant fasta file was generated by manually changing original residues in 1TSR.A fasta file by those that are the result of mutation; (iii) PSI-BLAST as alignment method; (iv) 70% of identity. The results are available to be explored and analyzed in VERMONT[1].

Complex network centrality measures for mutations Arg273His (no structural effects) and Ile195Thr (highly destabilising), discussed in the main paper, are presented in Figures 3 and 5, respectively.

Gly245Ser mutation corresponds to position 152 in the structural alignment, and it is non-conservative as Gly is nonpolar aliphatic and Ser is polar neutral. This column is highly conserved in the structural alignment, as Gly is present at 94% of the proteins. The accessibility is conserved and has low values (3 up to 48.6), as the whole column presents the same shade of gray. With regard to the topological properties, the degree is conserved (2 to 5); the betweenness is low (light shades in the column) and not well conserved, as the color is not very similar in the whole column; closeness is relatively conserved. Inspecting the interactions established in the alignment position 152, we see there are only hydrogen bonds, except in the PDB 2BIO.A that also presents a hydrophobic interaction. Considering all these aspects, we tend to point this mutation as probably damaging as it is non-conservative and has low and conserved values for accessibility, which means residues in this position are not exposed to solvent, being in the protein core, where we believe a mutation tends to have more impact on protein stability. This conclusion is in accordance with FoldX, which outlines this position with a red rectangle.

Arg249Ser, which is represented at position 156 in the structural alignment, is a non-conservative mutation as Arg is polar positive and Ser is polar neutral. The position 156 is highly conserved in the structural alignment as 90% of the residues are Arg. The accessibility is conserved and relatively low (9.5 up to 41.9)

[1]http://bioinfo.dcc.ufmg.br/vermont/results/view/case_study1/alignment

with a shade of gray in the whole column. Inspecting the topological properties, the degree and betweenness are not well conserved, as the column does not present a homogeneous shade; closeness is relatively conserved. Regarding the interactions, about 90% of the residues establish charged interactions, of which all are Arginines. Hydrogen bonds are highly conserved, being established by 99% of the residues, while hydrophobic interactions are relatively conserved in this column as 64% of the residues established this interaction type. Although Serine is also able to establish hydrogen bonds, the high conservation of charged interactions in this column indicates that Arginine likely further stabilize the protein. Thus, we would point this mutation as likely damaging because it is non-conservative, with low and conserved accessibility, despite FoldX points out this mutation as neutral.

Arg248Ala, which is represented in the structural alignment position 155, is a non-conservative mutation as Arg is polar positive and Ala is nonpolar aliphatic. This column is highly conserved in the structural alignment, presenting only Arginines. The accessibility is relatively high (27.3 up to 89.4) and conserved, with the whole column in a light shade of blue. With regard to the topological properties, the degree is well conserved (values 2 and 4); betweenness is not conserved; closeness is relatively conserved. When it comes to the interactions in position 155, all residues establish hydrogen bonds, so this interaction is highly conserved. Charged attractive interactions are not conserved as only 1 residue establishes this type of interaction. It is noteworthy that this mutation occurs in the DNA binding site (Figure 6), therefore the Arg248Ala mutation would likely diminish the protein-DNA affinity. Therefore, we consider this mutation as probably damaging due to its position, what is also confirmed by the high frequency of Arginines in this column. Bearing this in mind, we believe FoldX pointed out such mutation as neutral because it did not take the binding site into consideration.

Cys242Ser mutation corresponds to structural alignment position 149, and it is non-conservative as Cys is a residue with special properties (it can establish disulfide bridge) and Ser is polar neutral. The position 149 is highly conserved in the structural alignment with Cysteine residues. There is only one row, PDB id 2P52.A, that presents Ser (S). The accessibility is conserved and present low values (7.6 up to 38.6) having a light shade of gray, the only exception being 2P52.A (accessibility 62.7), which we consider as an outlier. Considering the topological properties, degree is well conserved (2 up to 5); betweenness is not conserved; closeness is relatively conserved. Regarding the interactions of alignment position 149, all residues establish hydrogen bonds, which are highly conserved, and 32 residues establish hydrophobic interaction. Having these aspects in mind, we consider this mutation as damaging as it is non-conservative (changing a cysteine, which is a residue with special properties) and it occurs in a position with low and conserved accessibility. On the other hand, FoldX points this mutation as slightly stabilizing. We further investigated Cys242 and discovered that it helps to stabilize p53 through a coordination system together with Zinc, Histidine and two other Cysteines [4, 5]. Therefore, Cys242Ser mutation is indeed destabilizing.

His168Arg mutation is represented in the structural alignment position 75, and it is conservative as both residues are polar positive. The alignment position 75 seems highly conserved, as 93% of the residues are Histidines, and the remaining residues are Arginines. The accessibility is relatively low and conserved (5.1 up to 39.8). Regarding the topological properties, the degree is relatively conserved (3 up to 7); betweenness is not well conserved; closeness is relatively conserved, being in a region with a light shade of yellow. When it comes to the interactions of position 75, all residues establish hydrogen bond interactions, while 82%, 85% and 87% of the residues establish charged attractive, charged repulsive and hydrophobic interactions, respectively, which are well conserved. Although FoldX points out this mutation as neutral, we consider this mutation as likely damaging because accessibility is relatively low and conserved, and the interactions are well conserved. As showed in [5], the Histidine substitution produced a distortion around the mutation site, what caused the residues 166-170 to be omitted in the solved structure (PDB 2BIN) (Figure 7). The authors also showed that the combination of both His168Arg and Arg249Ser mutation reversed the structural changes induced by these single mutations. In fact, all Arginines we observed in the position 75 appeared only when the Arg249Ser mutation occurred (position 156), what further confirms that the single His168Arg mutation is damaging.

Val143Ala, which is in the position 50 in the structural alignment, is conservative as Val and Ala are both nonpolar aliphatic. Column 50 is highly conserved, presenting only Valines, except 1 row (2J1W.A) that presents an Alanine. The accessibility is very low and conserved (0 up to 4.3). Considering the topological properties, degree is relatively conserved (3 up to 5); betweenness and closeness are relatively conserved. The hydrogen bonds and hydrophobic interactions in position 50 are highly conserved, as 100% and 91% of the residues, respectively, establish these interactions. Considering all these aspects, we tend to point out Val143Ala as damaging, because the position 50 presents very low and conserved accessibility

with highly conserved hydrophobic interactions, being a mutation in the protein core, which we believe have an impact on stability. Moreover, according to Lesk color scheme, the mutation is non-conservative, as Val is hydrophobic and Ala is small nonpolar. In fact, Val143Ala is a mutation which results in a residue with smaller volume (Ala). Our conclusion is in accordance with FoldX, which outlines this position with a red rectangle.

Availability of data and material
Vermont interactive platform and *Additional file 1* are available at:
http://bioinfo.dcc.ufmg.br/vermont/

Competing interests
The authors declare that they have no competing interests.

Funding
This work has been supported by Coordenação de Aperfeiçoamento de Pessoal de Nível Superior (CAPES), Conselho Nacional de Desenvolvimento Científico e Tecnológico (CNPq) and Fundação de Amparo à Pesquisa do Estado de Minas Gerais (FAPEMIG).

Author's contributions
SAS and RCM conceived the VERMONT platform. AVF and PMM designed and implemented the tool. SSG, SSA, and VSR implemented algorithms for property computation. SAS and RCM analyzed the results and wrote the manuscript. All authors read and approved the final manuscript.

Author details
[1] Department of Computer Science, Universidade Federal de Minas Gerais, Belo Horizonte, Brazil. [2] Department of Biochemistry and Immunology, Universidade Federal de Minas Gerais, Belo Horizonte, Brazil. [3] Computer Science Department, Universidade Federal de Viçosa, Viçosa, Brazil.

References
1. Cheng, T.M., Lu, Y.-E., Vendruscolo, M., Blundell, T.L., *et al.*: Prediction by graph theoretic measures of structural effects in proteins arising from non-synonymous single nucleotide polymorphisms. PLoS Comput Biol **4**(7), 1000135 (2008)
2. Li, Y., Wen, Z., Xiao, J., Yin, H., Yu, L., Yang, L., Li, M.: Predicting disease-associated substitution of a single amino acid by analyzing residue interactions. BMC bioinformatics **12**(1), 14 (2011)
3. Energy range. https://evosite3d.blogspot.com.br/2015/03/tutorial-estimating-stability-effect-of.html?m=1
4. Friedler, A., Veprintsev, D.B., Hansson, L.O., Fersht, A.R.: Kinetic instability of p53 core domain mutants implications for rescue by small molecules. Journal of Biological Chemistry **278**(26), 24108–24112 (2003)
5. Joerger, A.C., Ang, H.C., Veprintsev, D.B., Blair, C.M., Fersht, A.R.: Structures of p53 cancer mutants and mechanism of rescue by second-site suppressor mutations. Journal of Biological Chemistry **280**(16), 16030–16037 (2005)
6. Pettersen, E.F., Goddard, T.D., Huang, C.C., Couch, G.S., Greenblatt, D.M., Meng, E.C., Ferrin, T.E.: UCSF Chimera–a visualization system for exploratory research and analysis. J Comput Chem **25**(13), 1605–1612 (2004)

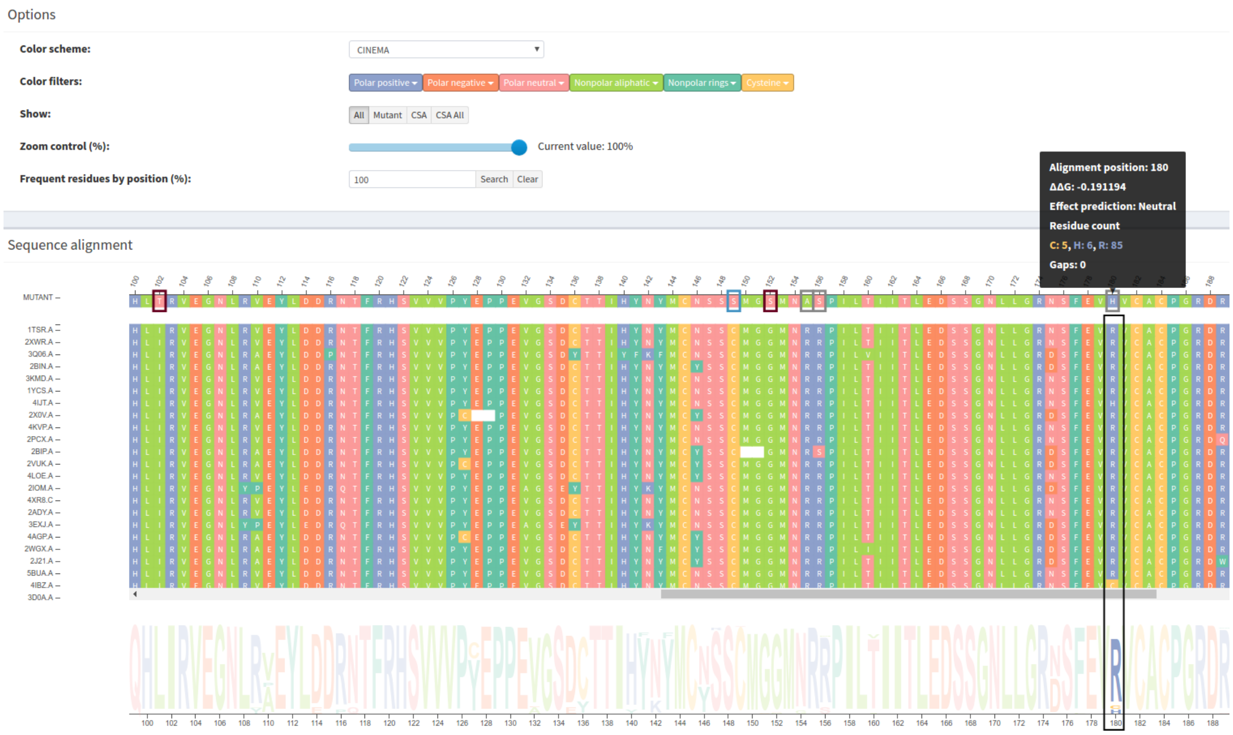

Figure 2 *Residue conservation highlighted for alignment position 180, which corresponds to the conservative mutation Arg273His in protein p53 (1TSR.A). This position is highly conserved using the CINEMA color scheme, with about 89% of Arg and 5% of His and Cys each.*

Table 1 Mutations (nsSNPs) in the p53 (PDBid 1TSR) core domain that were experimentally characterized.

Mutant categories	Mutations
No structural effects	Arg273His
Weakly/locally destabilising	Gly245Ser<br>Arg249Ser<br>Arg248Ala
Highly destabilising/global unfolding	Cys242Ser<br>His168Arg<br>Val143Ala<br>Ile195Thr

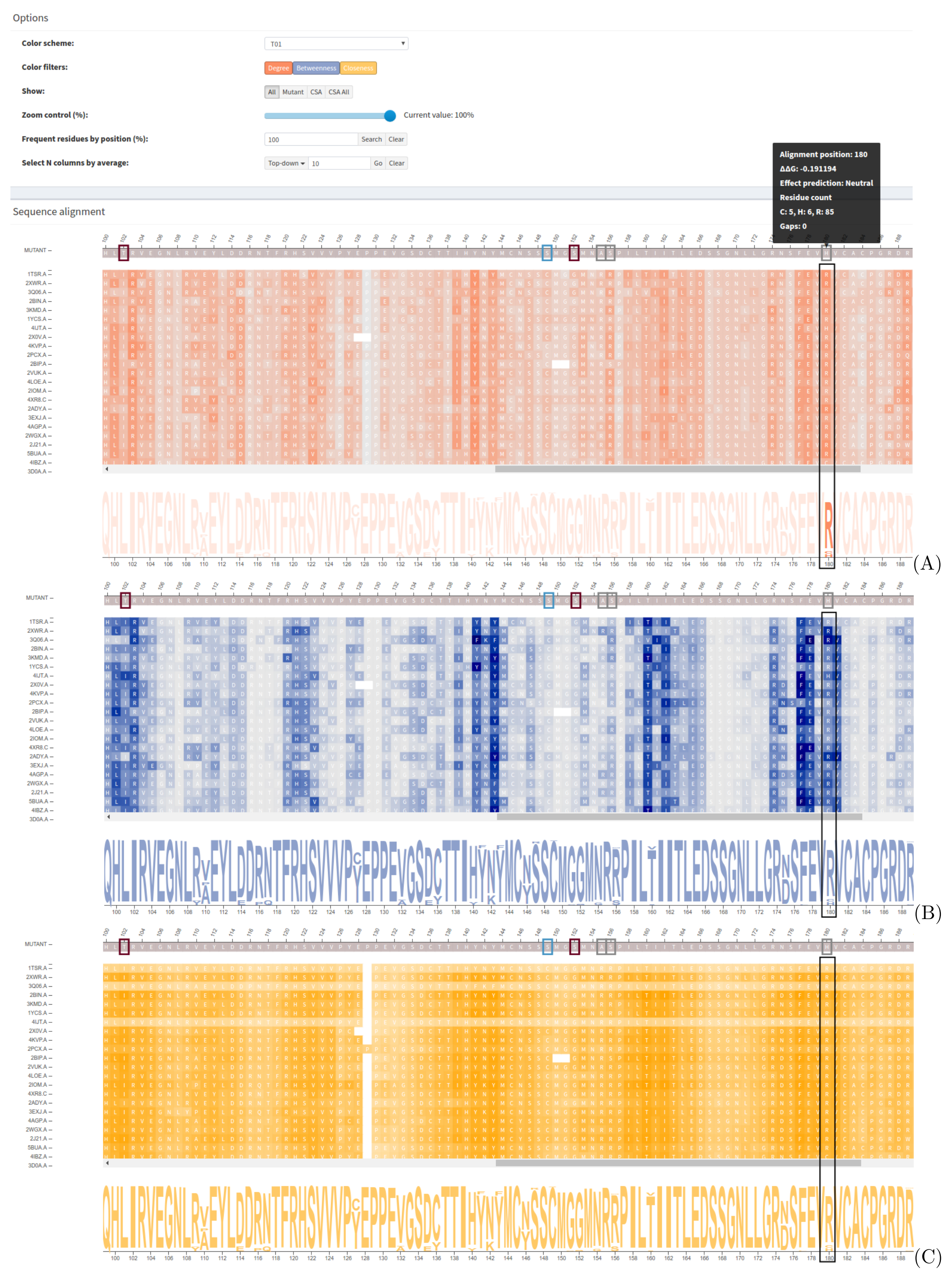

Figure 3 *Topological properties (network centrality measures) highlighted for alignment position 180, which corresponds to mutation Arg273His in protein p53 (1TSR.A). (A) Degree heatmap. Degree is conserved as the highlighted column presents a similar shade of orange. (B) Betweenness heatmap. Column 180 presents many different shades of blue, which means that betweenness is not conserved. (C) Closeness heatmap. This measure is relatively conserved as column 180 presents quite similar shades of yellow. Note that closeness has conserved regions.*

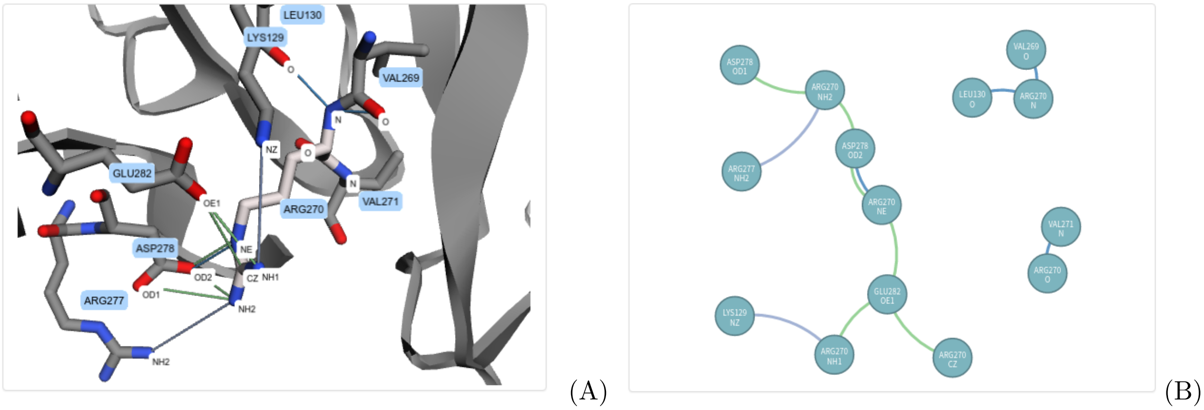

Figure 4 Interactions established by residue Arg270 of 3EXL.A. This residue is in the alignment position 180, which corresponds to mutation Arg273His in protein p53 (1TSR.A). By clicking on any residue on the *Interactions module*, VERMONT shows its interactions in a 3D molecule viewer and in a 2D graph. Here we illustrate how the domain specialist can inspect the interactions established by each particular residue in a detailed manner in the atomic level. (A) 3D interactions for Arg270 of 3EXL.A in a molecule viewer. (B) 2D graph schematic representation for interactions from Arg270 of 3EXL.A.

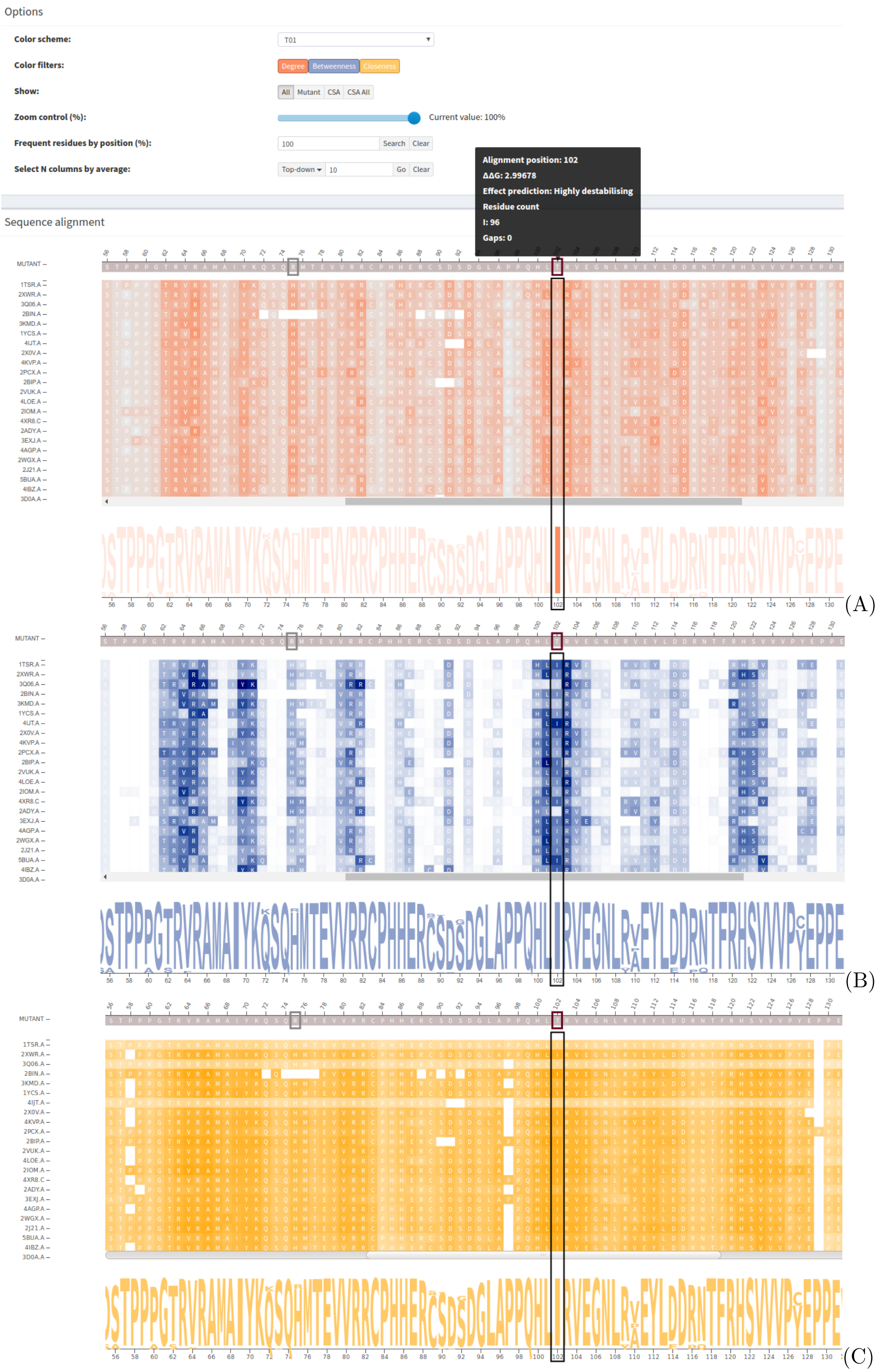

Figure 5 *Topological properties (network centrality measures) highlighted for alignment position 102, which corresponds to mutation Ile195Thr in protein p53 (1TSR.A). (A) Degree heatmap. Degree is relatively conserved as the highlighted column presents a similar shade of orange. (B) Betweenness heatmap. Betweenness is not conserved as column 102 presents many different shades of blue. (C) Closeness heatmap. This measure presents relative conservations in column 102 as we see similar shades of yellow, but there are some positions in this colum that presents very low values for closeness (light sade of yellow).*

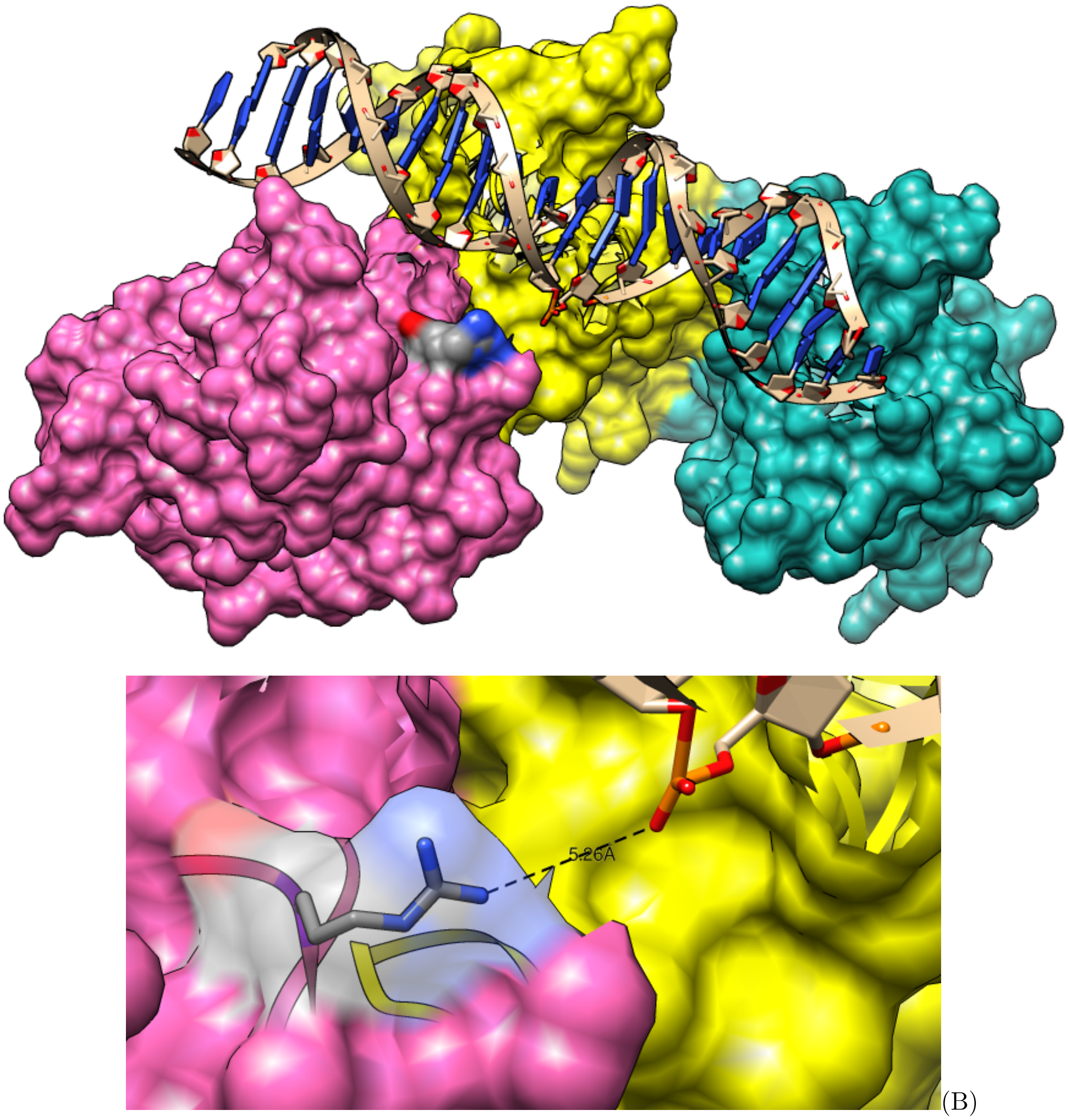

(A)

(B)

Figure 6 *Protein-DNA interface in PDB 1TSR. (A) DNA binding overview. Chain A surface is shown in purple and Arg248 is colored by chemical elements. (B) Probable charged attractive interaction between Arg248 and the DNA. Figures generated with Chimera [6].*

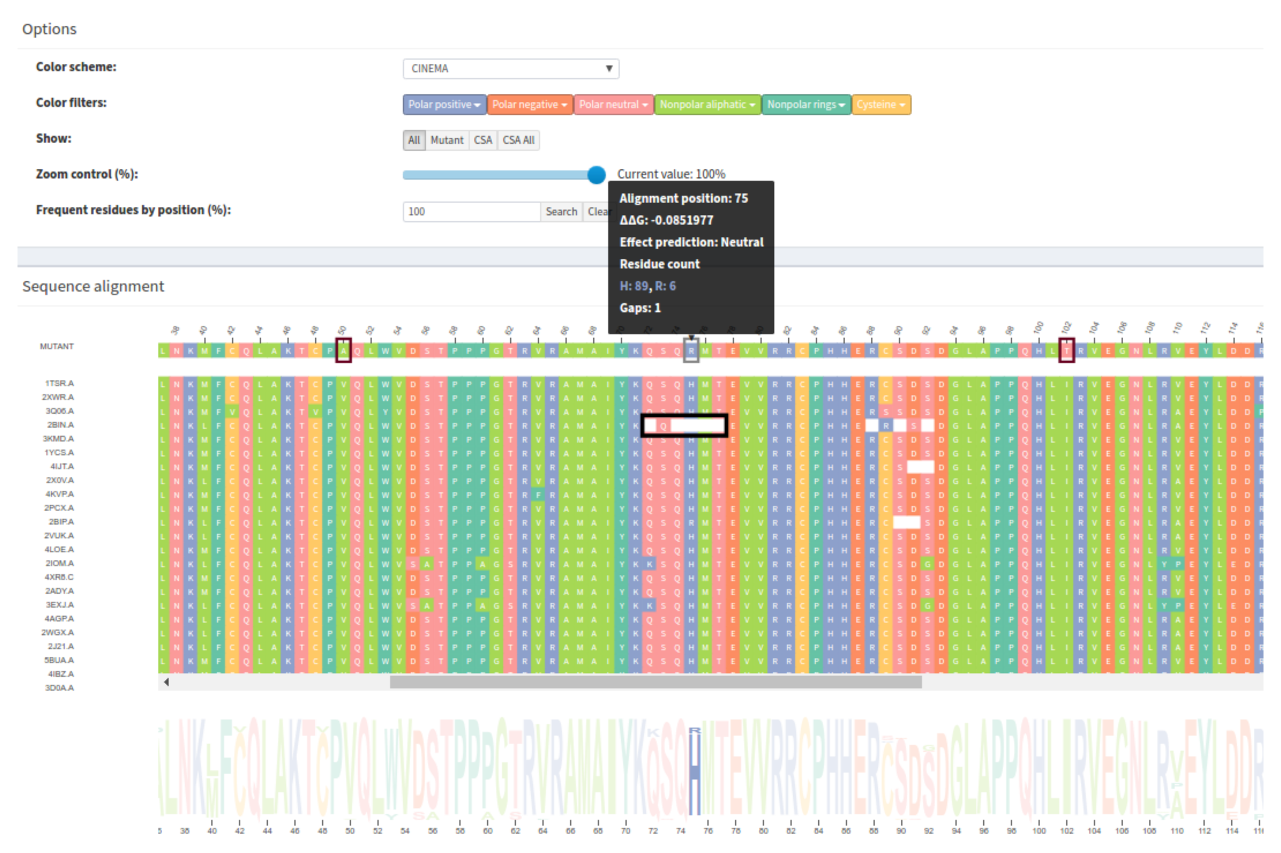

Figure 7 *Residue conservation highlighted for alignment position 75, which corresponds to the conservative mutation His168Arg in protein p53 (1TSR.A), and missing residues (166-170), which were experimentally observed for His168Arg mutation (PDB 2BIN), highlighted by the black square.*
